# Incidental Risk of Type 2 Diabetes Mellitus among Patients with Confirmed and Unconfirmed Prediabetes

**DOI:** 10.1371/journal.pone.0157729

**Published:** 2016-07-18

**Authors:** Kimberly D. Brunisholz, Elizabeth A. Joy, Mia Hashibe, Lisa H. Gren, Lucy A. Savitz, Sharon Hamilton, Wayne Cannon, Jaewhan Kim

**Affiliations:** 1 Institute for Healthcare Delivery Research, Intermountain Healthcare, Salt Lake City, Utah, United States of America; 2 Division of Public Health, School of Medicine, University of Utah, Salt Lake City, Utah, United States of America; 3 Primary Care Clinical Program, Intermountain Healthcare, Salt Lake City, Utah, United States of America; 4 Office of Research, Intermountain Healthcare, Salt Lake City, Utah, United States of America; Weill Cornell Medical College in Qatar, QATAR

## Abstract

**Objective:**

To determine the risk of type 2 diabetes (T2DM) diagnosis among patients with confirmed and unconfirmed prediabetes (preDM) relative to an at-risk group receiving care from primary care physicians over a 5-year period.

**Study Design:**

Utilizing data from the Intermountain Healthcare (IH) Enterprise Data Warehouse (EDW) from 2006–2013, we performed a prospective analysis using discrete survival analysis to estimate the time to diagnosis of T2DM among groups.

**Population Studied:**

Adult patients who had at least one outpatient visit with a primary care physician during 2006–2008 at an IH clinic and subsequent visits through 2013. Patients were included for the study if they were (a) at-risk for diabetes (BMI ≥ 25 kg/m2 and one additional risk factor: high risk ethnicity, first degree relative with diabetes, elevated triglycerides or blood pressure, low HDL, diagnosis of gestational diabetes or polycystic ovarian syndrome, or birth of a baby weighing >9 lbs); or (b) confirmed preDM (HbA1c ≥ 5.7–6.49% or fasting blood glucose 100–125 mg/dL); or (c) unconfirmed preDM (documented fasting lipid panel and glucose 100–125 mg/dL on the same day).

**Principal Findings:**

Of the 33,838 patients who were eligible for study, 57.0% were considered at-risk, 38.4% had unconfirmed preDM, and 4.6% had confirmed preDM. Those with unconfirmed and confirmed preDM tended to be Caucasian and a greater proportion were obese compared to those at-risk for disease. Patients with unconfirmed and confirmed preDM tended to have more prevalent high blood pressure and depression as compared to the at-risk group. Based on the discrete survival analyses, patients with unconfirmed preDM and confirmed preDM were more likely to develop T2DM when compared to at-risk patients.

**Conclusions:**

Unconfirmed and confirmed preDM are strongly associated with the development of T2DM as compared to patients with only risk factors for disease.

## Introduction

Type 2 diabetes mellitus (T2DM) is one of the most costly diseases due to the size of the population at risk and the fact that diabetes is a risk factor for almost all other chronic diseases[1,2]. The World Health Organization has predicted a global increase in diabetes prevalence by 39% between the years 2000 and 2030, representing a global increase to 366 million people by the year 2030[3].In additional to the millions of individuals with T2DM, there are an estimated 86 million Americans identified with prediabetes (preDM) who are at increased risk for the development of T2DM over time, yet only 14% of these individuals are aware of their condition [4].

Two groups of patients have emerged in the recent literature with the highest susceptibility of being associated with incident diabetes: (a) at-risk patients defined by American Diabetes Association (ADA) criteria, including body mass index (BMI) ≥25 kg/m^2^ and one additional risk factor: high risk ethnicity, first degree relative with diabetes, elevated triglycerides or blood pressure, low HDL, diagnosis of gestational diabetes or polycystic ovary syndrome, or birth of a baby weighing >9 lbs); and (b) patients who meet the preDM criteria through laboratory testing of HbA1c (A1c 5.7–6.49%) or fasting plasma glucose (FPG 100–125 mg/dL) [4–6].

A handful of studies have shown that a quarter of those with confirmed preDM will develop diabetes within 3 to 5 years of detection [7]. Observational evidence suggests that there is an association between confirmed preDM and complications of diabetes such as early nephropathy, small fiber neuropathy, early retinopathy, and risk of macrovascular disease [8]. Beginning in the early 1990s, several prominent studies have demonstrated that strategies to support weight loss and weight loss maintenance are the key to preventing development of T2DM in patients with preDM or those with risk factors for disease [9–14].

Patients who have undiagnosed preDM or unconfirmed preDM, have also been identified by the ADA as a vital target population; yet to date, there has been little data collected on how to identify this population and the trajectory of illness that a patient with unconfirmed preDM might face as compared to other risk groups [15]. As postulated by this study, unconfirmed preDM patients were identified by pairing laboratory studies that are routinely ordered in clinical practice: a fasting lipid panel accompanied by a chemistry panel on the same day that documents a glucose level between 100–125 mg/dL. Patients meeting this “unconfirmed” criteria may not have any evidence of preDM documented in their medical record since their provider was unaware of their condition, and furthermore, these patients may not have been treated using evidence-based therapies such as metformin to impede disease progression. Typically when clinicians intuitively suspect a greater risk of T2DM they will order laboratory tests such as FPG or HbA1c to confirm their suspicions. However, for patients who are not yet on their radar, an elevated fasting glucose from a chemistry panel ordered while evaluating or screening for conditions such as hypertension or heart disease may be indicative of increasing T2DM risk, similar to those screened with an A1c or FPG.

Because the size of the population at-risk for developing diabetes is a substantial proportion of the patients we serve, the need to prioritize patients for intervention based on severity of risk is necessary. By studying all patients with incremental risk for diabetes through differing identification phenotypes, the results of this study highlight which patients have the highest susceptibility for disease and should be intervened on first. Furthermore, the ability to assess laboratory-tested versus risk-factor based models of preDM identification has not been demonstrated. To address these gaps in knowledge, the primary purpose of this study is to determine if there is an incremental risk of T2DM among patients with confirmed preDM and unconfirmed preDM relative to an at-risk group receiving care from primary care physicians over a 5-year period.

## Methods

We utilized a longitudinal, closed cohort design to determine the association of T2DM over time among three different groups of patients considered at higher risk for disease.

### Study Subjects

The IH Enterprise Data Warehouse (EDW) identified a source population of adult patients (≥18 years of age) who had at least one outpatient visit with a primary care physician (family medicine, internal medicine, or geriatric specialty) during 2006–2008 and received continued treatment through 2013 (S1 file). The EDW collects routine clinical, administrative and financial information about individual patient encounters performed within the IH system (ie. contains data on all patients who receive medical care captured within our internal electronic health record from 22 hospitals and 185 outpatient clinics within the IH delivery system found across the state of Utah and southeast Idaho) and is accessible for projects related to quality improvement within the organization. Patients meeting source inclusion criteria were further delineated for study if they did not have a known death or prevalent T2DM. Patients who died during the study period were omitted due to any clinical differences suggestive of probable bias because of life-threatening medical conditions that would not typically be found within patients only at-risk for T2DM disease. As defined in S1 Table, we included patients for study if they met criteria for (a) at-risk for diabetes or (b) confirmed preDM or (c) unconfirmed preDM. The IH Institutional Review Board approved this study.

### Study Measurement

#### Baseline Characteristics

As determined by clinical health characteristics, patients with differing levels of disease were compared to assess whether differences in patient demographics, social factors, as well as clinical and practice characteristics existed prior to diagnosis. Baseline demographics included age, sex, and race/ethnicity. Clinical characteristics for the study cohort included the proportion of patients with chronic conditions prior to study enrollment, including: depression, coronary heart disease, congestive heart failure, atrial fibrillation, and high blood pressure. Criteria for chronic conditions are different for each condition; however, they are based on diagnosis codes and encounter data, and are approved by an internal expert committee of practicing providers (Table A in S1 Appendix). We also included the medication classes that were ordered (anti-hypertensive, atypical-neuroleptics, metformin, and statins) as well as weight (kilograms) and body mass index class (underweight, normal, overweight, or obese) at baseline. To adjust for practice variation, we attributed patients to a primary care provider and practice who provided the plurality of qualifying services (Current Procedural Terminology codes for outpatient office visit, preventive medicine visit, or wellness visit: 9920x, 9921x, 99385–87, 99395–97, G0101, G0402, G0438) in a given calendar year, with most recent service date breaking any ties. Data on potential practice confounders were collected at varying points in time when a patient touched the delivery system: the specialty type of primary care provider (family medicine, internal medicine, or geriatrics) and panel size of practice. We also included the geographical region where services were provided.

#### Study Endpoints

Time to diagnosis of T2DM was the primary outcome of interest. T2DM was defined by the National Committee for Quality Assurance (NCQA) through the Healthcare Effectiveness Data and Information Set (HEDIS) specifications [16–17]. These specifications require only one of the following to be met along with a diagnosis code of diabetes (ICD-9 code: 250): (a) two outpatient encounters on different dates of service; (b) one acute inpatient encounter; (c) one emergency department visit; or (d) patients who were dispensed insulin or hypoglycemic/anti-hyperglycemics on an ambulatory basis. Other outcomes included the number who converted to T2DM. We assessed all outcomes through December 31, 2013. For patients who were censored or did not develop T2DM disease, we used the last IH encounter as the censor date.

#### Statistical Analysis

We computed summary statistics which included means, medians, standard deviations, and ranges to describe the study population characteristics. We compared continuous variables between study groups using analysis of variance followed by adjustment for multiple comparisons using a Tukey pairwise analysis. We used Chi-square analysis to determine differences in proportions for categorical variables.

We utilized discrete survival analysis modeling to test the null hypothesis that time to T2DM diagnosis was no different among patients with differing levels of disease. We considered patients categorized as at-risk for diabetes the referent group. We generated hazard ratios after adjustment for static and time-varying variables, including demographic, clinical characteristics, and practice variation that are well-known to affect the risk of diabetes. Due to the intrinsically discrete intervals of interest for a provider, we divided the time-to-event data into intervals of 6-month increments and further adjusted the model for the number of times a patient visited the delivery system. We selected a 180-day interval of care because evidence-based guidelines suggest twice yearly follow up with a provider for patients at increased risk for T2DM [18]. We used non-adjusted Kaplan-Meier survival curves to visually compare T2DM risk across the groups.

We also assessed the effect of combining age and study group. We categorized patients into two age groups based on the screening recommendation from the American Diabetes Association[4–6]: (a) < 45 years and (b) ≥45 years. These age groupings, along with the study group, could be studied to determine the impact on time to T2DM.

For all analyses, we considered a *p*-value ≤.05 statistically significant, and analyzed all data using Stata 12.0 (Stata Corp, College Station, TX).

## Results

We identified 631,174 patients who received at least one outpatient visit with a primary care physician (family medicine, internal medicine, geriatric specialty) within the IH delivery system during 2006–2008 (see S1 Fig). Of these, we excluded 352,304 because they had no known increased risk for T2DM. We excluded an additional 213,138 patients because their age at time of study enrollment was <18 years of age. We excluded another 31,894 patients because they had a known date of death during the study. Of the study population that remained, we identified 8.76% (n = 33,838) patients as: at-risk (57.0%; n = 19,288), unconfirmed preDM (38.4%; n = 13,005) and those with confirmed preDM (4.6%; n = 1,545). For patients within the at-risk group, 100% had a body mass index ≥ 25 kg/m^2^; 37.4% were diagnosed with hypertension (blood pressure >140/90); 33.1% had an HDL <35mg/dL; 21.5% had triglycerides >250 mg/dL; 13.4% were of high-risk ethnicity; 2.9% had a baby weighing over >9lbs; 1.7% had a first degree relative with diabetes; 1.3% were diagnosed with gestational diabetes; and 0.7% were diagnosed with polycystic ovary syndrome (patients could have multiple indications for risk, and thus the proportion among the group does not add up to 100%).

Baseline demographic and clinical characteristics are summarized in S2 Table. Over half (59.38%) of the unconfirmed preDM group were male, compared to 51.01% and 48.87% in the at-risk and confirmed preDM group. Patients tended to be older in both the unconfirmed and confirmed preDM groups as compared to the at-risk group (54.1, 54.1, and 48.7 years, respectively).

Patients with confirmed preDM tended to have more depression, coronary heart disease, congestive heart failure, atrial fibrillation, and high blood pressure (*p* <.001) as compared to patients in the other groups. Similarly, patients with confirmed preDM tended to have more ordered medications at time of diagnosis (*p* < .001) as compared to other study groups. Weight at baseline did not seem to differ clinically; however, the finding was statistically significant. Patients in the unconfirmed and confirmed preDM group tended to be categorized more commonly as obese rather than those at-risk (66.01%, 66.93%, and 52.14%; *p* < .001).

Actuarial risk for T2DM is shown in S2 Fig, demonstrating an increasing separation between the study groups across the entire study period (*p* < .001). There was also a significant difference at 3- and 5-year intervals for risk of T2DM when comparing the study groups *(p* < .001). Overall 9% (n = 2,883) had converted to T2DM within 5 years, 20% (n = 302) in the confirmed preDM group, 11% (n = 1,391) in the unconfirmed group, and 6.0% (n = 1,190) in the at-risk group. The average study follow-up did not seem to differ clinically among the confirmed, unconfirmed and at-risk groups (4.9 years, 5.1 years, and 5.2 years; *p* < .001); however, the finding was statistically significant. A sensitivity analysis was performed when a group of healthy patients, excluded from the main analysis, (criteria: no known risk of T2DM; at least 2 encounters to their provider during 2006–2008; no known death or prevalent T2DM) were included as the referent group. There were no significant differences in terms of the incident risk of developing T2DM over time (Fig A in S2 Appendix).

Utilizing discrete survival analyses adjusted for possible confounders, patients with unconfirmed preDM were 67% more likely to develop T2DM as compared to those at-risk (HR 1.67; CI 1.53, 1.83; *p* < .001). Patients with confirmed preDM had over a 2.5fold increase of incident T2DM as compared to at-risk patients (HR 2.73; CI 2.37, 3.15; *p* < .001). Patients on metformin (HR 4.01; CI 3.37, 4.78; *p* < .001) and those with a diagnosis of high blood pressure at study enrollment (HR 1.16; CI 1.05, 1.27; *p* = .002) tended to have significantly greater risk of developing T2DM while patients with depression showed a decreased risk of disease (HR 0.85; CI 0.77, 0.94; *p* = .001). Patients with a BMI that was considered either overweight or obese were at higher risk for T2DM (*p* < .001). All multivariate results are documented in S3 Table. Also, when assessing age and study group together, there was a significant association with developing T2DM among those with older age and confirmed preDM (S4 Table).

## Discussion

Although a sizeable proportion of the study population did not develop incident disease over the 5-year study period, patients with confirmed preDM demonstrated a compelling difference (greater than a 2.5-fold increase) in the development of diabetes, while patients with unconfirmed preDM had more modest increases in disease progression when compared to patients with risk factors. In a meta-analysis of prospective studies published between 1979 and 2004, annualized incidence rates of progression to diabetes in patients with various categories of glucose intolerance were comparable (15–25%) to results seen in this study [19]. In subsequent major studies, annual progression estimates were also similar: 11% in the Diabetes Prevention Program (DPP) outcomes study which randomized preDM patients to receive intensive lifestyle therapy, metformin, or placebo [20,21] and 9% in participants with impaired fasting glucose and 7% in those with HbA1c between 5.7–6.4% enrolled in a Japanese population-based study [21].

The incidence rates detected within this study are clinically concerning and will be financially devastating to not only our transforming delivery system, but also to the patients and their families as we move into an accountable environment for care delivery. In the study, the majority of participants had not even reached a Medicare eligible age (usual age of eligibility is ≥65 years), demonstrating that those who develop chronic diseases will have to live with their disease for many years to come. In a series of rigorous cost analyses conducted over the past decade, the American Diabetes Association estimated that Americans with diagnosed diabetes have annual medical expenditures that are $7,900 more, or approximately 2.3 times higher, than they would be in the absence of diabetes ($13,700 vs. $5,800) [4]. Therefore, it is important to identify not only the triggers that predispose progression but also potential interventions that could impede or slow incident disease over time.

Earlier identification of patients with preventable disease who have the greatest risk and/or may benefit from intervention is one mechanism for redesigning healthcare within a transforming delivery system. While a large body of literature supports the effectiveness of intervening on a population of patients with a confirmed diagnosis of preDM or those with identifiable risk factors for disease, much less evidence exists on ways to identify patients with preDM who do not know they have the condition and if their risk profile is similar to other groups [20, 22–23]. Clinicians who suspect a greater risk of T2DM can order laboratory tests such as FPG or HbA1c to confirm their suspicions. However, a significant number of patients (n = 13,005 in our study) are not yet on their radar, but were found to have elevated FPG from chemistry panels ordered while evaluating or screening for other conditions. This confirms our findings that patients with a confirmed diagnosis of preDM demonstrated an incremental risk of developing T2DM in comparison to patients with only risk factors for disease, more so than those with unconfirmed preDM. Yet, identifying patients at greatest risk or those with the largest benefit (ie. such as those with confirmed preDM) will be one mechanism to manage the population’s health in the future to delay or avoid diagnosis of T2DM.

The findings of this study clearly support previous work that demonstrate the increased risk of incident T2DM disease over time for patients with confirmed preDM, yet also contributes to a limited body of knowledge surrounding which groups of patients with established diabetes risk should be intervened on first and subsequently, uncovering data methods that can be used to recognize patients with un-identified disease. It should be noted that while confirmation of preDM increases the risk of T2DM, we also confirmed that increasing age also independently increases a person’s chance of diagnosis. This finding lends evidence to the ADA criteria that recommend screening for T2DM and preDM in otherwise healthy individuals ≥45 years of age at least every 3 years[4,24]. Patients with depression at study entry seemed to have a protective effect for developing T2DM, which may be a mechanism of high functioning, multidisciplinary primary care teams to guide patients to care faster. This finding warrants further study.

### Limitations

We carefully selected the study groups according to criteria found within the literature, but there may be inherent unaccounted differences due to data miscoding or selection bias that still remain, affecting the results observed. Patients within the confirmed and unconfirmed preDM groups only had one documented laboratory test, although most clinical experts will require at least two tests to confirm suspicions of preDM. Patients within the at-risk study group may have variation in the ability to identify risk factors. When possible, we used validated IH registries to identify risk among patients (i.e., hypertension, polycystic ovary syndrome, gestational diabetes, and birth weights >9lbs); however, the ability to identify patients with family history of diabetes may be more difficult to classify because it relies on patient self-report and providers to document this in the medical record. We excluded patients from the study with a prevalent T2DM diagnosis, yet there remains a possibility that a patient’s diagnosis was missed within our data systems due to care that was delivered outside of the IH system. The percentage of patients who were lost to follow-up and encounters that occurred outside of the IH delivery system was not defined or captured within the data in this study; however, IH does encompass roughly 60% of the care delivered within the state of Utah. Additionally, patients with a known death were excluded to eliminate any probable bias of any life-threatening medical conditions that would not typically be found within patients only at-risk for T2DM disease. Since the IH Diabetes Registry does not distinguish between Type 1 or ype 2 diabetes mellitus, our primary outcome may still include both types of the disease. To account for our inability to distinguish between Type 1 or Type 2 diabetes mellitus within the data, we excluded pediatric patients from the study population, limited the provider population to only primary care providers, and did not consider those who delivered specialty care typically reserved for patients with type 1 diabetes, such as endocrinologists and diabetologists. Female patients who consider their obstetrician or gynecologist as the provider who delivers their care primarily were not studied in this analysis and may warrant further study to determine their risk of T2DM. While the methodology used in this study attempted to account for practice variation across the IH clinics where the patients received care, it might not account for all variation in practice which could affect the observed results. It should also be acknowledged that the study population was largely Caucasian and may not be generalizable to populations outside of IH (~86% within this study compared to ~78% nationwide). Patients from outside of the state of Utah do access the delivery system, yet are among the minority of IH encounters. Social determinants of health such as where the patient was born, their current living conditions, and education and income level have also been associated with health outcomes, but were not available for study.

### Conclusions

Patients with unconfirmed and confirmed preDM had a higher risk of T2DM as compared to patients with only risk factors for disease. While early identification and risk stratification of T2DM is indispensable, the real opportunity lies with improving the awareness of the condition within our communities—empowering patients with the knowledge of their risk status to activate them as partners in a culture of health. Coupling screening and awareness of preDM with team-based care delivery and payment reform geared towards value and service will only emphasize proactive identification and assessment for those at-risk for chronic disease progression and improve the overall health for most targeted populations, not just for patients at-risk for T2DM.

## Supporting Information

S1 Appendix Table A. Definitions of chronic conditions.
(DOCX)

S2 Appendix Fig A. Kaplan-Meier actuarial survival curve showing accumulated diabetes diagnosis rates over time among patients with confirmed and unconfirmed prediabetes, those at-risk for diabetes, relative to a group with no identified diabetes risk.
(DOCX)

S1 Fig Study criteria for inclusion. (DOCX)

S2 Fig Kaplan-Meier actuarial survival curve showing accumulated diabetes diagnosis rates over time among study groups. (DOCX)

S1 File Deidentified_Incidental risk of T2DM 01 25 2016.dta. (DTA)

S1 Table Definition of study groups. (DOCX)

S2 Table Baseline Characteristics. (DOCX)

S3 Table Incidental risk of type 2 diabetes mellitus among patients with confirmed and unconfirmed prediabetes as compared to those at-risk for disease. (DOCX)

S4 Table Incidental risk of type 2 diabetes mellitus associated with the study group and age greater or less 45 years of age among study patients. (DOCX)
