# Supplementary material for: Incidental Risk of Type 2 Diabetes Mellitus among Patients with Confirmed and Unconfirmed Prediabetes
Source: PLoS One. 2016 Jul 18;11(7):e0157729. doi: 10.1371/journal.pone.0157729 (PMC4948775; doi:10.1371/journal.pone.0157729)
Supplement: S1 Appendix — Table A. Definitions of chronic conditions. (DOCX) [file pone.0157729.s001.docx]

S1 Appendix: Definitions of chronic conditions

Table A

| **Chronic Condition** | **Diagnoses (ICD-9-CM)*** | **Encounters (CPT)*** | **Exclusions** |
| --- | --- | --- | --- |
| High Blood Pressure | 360.42, 362.11, 401, 401.0, 401.1, 401.9, 402, 402.0, 402.00, 402.01, 402.1, 402.10, 402.11, 402.9, 402.90, 402.91, 403, 403.0, 403.00, 403.1, 403.10, 403.9, 403.90, 404, 404.0, 404.00, 404.01, 404.1, 404.10, 404.11, 404.90, 404.9, 404.91, 405, 405.0, 405.01, 405.09, 405.1, 405.11, 405.19, 405.9, 405.91, 405.99, 437.2 | Outpatient visit with either: 99201-05, 99211-15, 99241-45, 99341-50, 99381-87, 99391-97, 99401-04, 99411-12, 99420, 99429, 99455-56 | No documentation of renal transplant |
| Atrial Fibrillation | 427.31 | Inpatient admission with either: 3734, 3726-28 | none |
| Coronary Artery Disease | 410.xx, 411.0, 411.1, 411.81, 411.89, 412.0, 413.0, 413.9, 414.0, 414.01, 414.02, 414.03, 414.04, 414.05, 414.06, 414.07, 414.11, 414.80, 414.90 | none | none |
| Heart Failure | 398.91, 402.01, 402.11, 402.91, 404.01, 404.03, 404.11, 404.13, 404.91, 404.93, 428.0, 428.1, 428.20, 428.21, 428.22, 428.23, 428.30, 428.31, 428.32, 428.33, 428.40, 428.41, 428.42, 428.43, 428.9 | none | none |
| Depression | 296.2, 296.20, 296.21, 296.22, 296.23, 296.24, 296.25, 296.26, 296.3, 296.30, 296.31, 296.32, 296.33, 296.34, 296.35, 296.36,  296.82, 296.90, 298, 298.0, 300.4, 309.1, 309.28, 311 | Hospital admission *or...* | none |
|  |  | Emergency Department Visit *or…* |  |
|  |  | Outpatient visit with either: 99201-05, 99211-15, 99241-45, 99341-50, 99381-87, 99391-97, 99401-04, 99411-12, 99420, 99429, 99455-56 |  |
| ICD-9-CM: The International Classification of Diseases, Ninth Revision, Clinical Modification; CPT: Current Procedural Terminology | | | |
| *To be identified with a chronic condition, specifications require at least one CPT and ICD-9-CM code to be paired on the same day | | | |
