# Supplementary material for: Incidental Risk of Type 2 Diabetes Mellitus among Patients with Confirmed and Unconfirmed Prediabetes
Source: PLoS One. 2016 Jul 18;11(7):e0157729. doi: 10.1371/journal.pone.0157729 (PMC4948775; doi:10.1371/journal.pone.0157729)
Supplement: S1 Fig — (DOCX) [file pone.0157729.s003.docx]

S1 Figure: Study criteria for inclusion

Source Population:

n= 631,174

Received care from IH PCP between 2006-2008

597,336 Excluded:

352,304 no known T2DM risk

213,138 <18 years at enrollment

31,894 had death during study

Study Population:

n=33,838

Unconfirmed Prediabetes:

n=13,005

Confirmed Prediabetes:

n=1,545

At-risk for Diabetes:

n=19,288
