# Supplementary material for: Incidental Risk of Type 2 Diabetes Mellitus among Patients with Confirmed and Unconfirmed Prediabetes
Source: PLoS One. 2016 Jul 18;11(7):e0157729. doi: 10.1371/journal.pone.0157729 (PMC4948775; doi:10.1371/journal.pone.0157729)

S2 Figure: Kaplan-Meier actuarial survival curve showing accumulated diabetes diagnosis rates over time among study groups.


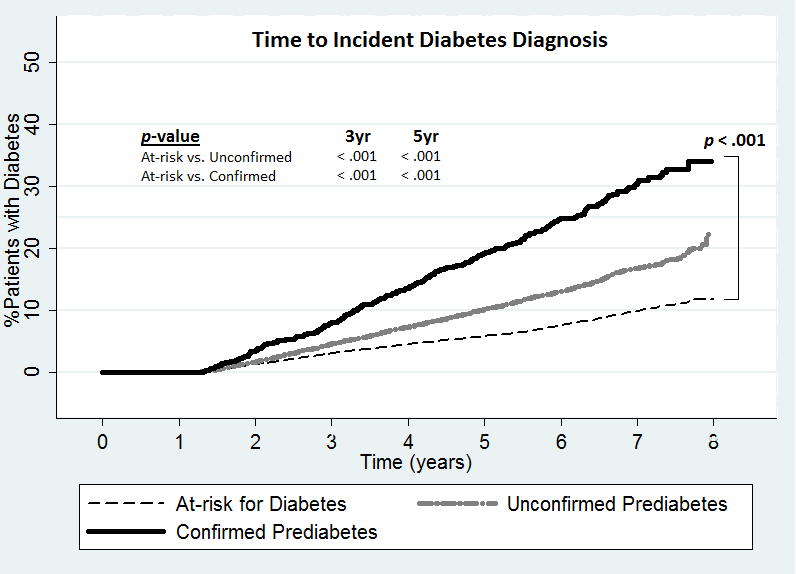

Supplement: S2 Fig — (DOCX) [file pone.0157729.s004.docx]
