# Supplementary material for: Incidental Risk of Type 2 Diabetes Mellitus among Patients with Confirmed and Unconfirmed Prediabetes
Source: PLoS One. 2016 Jul 18;11(7):e0157729. doi: 10.1371/journal.pone.0157729 (PMC4948775; doi:10.1371/journal.pone.0157729)
Supplement: S1 Table — (DOCX) [file pone.0157729.s006.docx]

S1 Table: Definition of study groups

| Study Group: | Definition: |
| --- | --- |
| At-risk for Diabetes | BMI ≥ 25 kg/m2 + one additional risk factor:   - High risk ethnicity (Asian, African Americans, Hispanic, Native Americans) - 1^st^ degree relative with Diabetes - HDL <35 mg/dL - Triglycerides >250 mg/dL - Hypertension >140/90 mmHg - Gestational Diabetes diagnosis - Polycystic Ovary Syndrome diagnosis - Baby weighing >9lbs |
| Unconfirmed Prediabetes | Chemistry Panel (with Glucose 100-125 mg/dL) on same day as Fasting Lipid Panel |
| Confirmed Prediabetes | HbA1c 5.70-6.49%  *or.....*  Fasting Plasma Glucose 100-125 mg/dL |
