# Supplementary material for: Incidental Risk of Type 2 Diabetes Mellitus among Patients with Confirmed and Unconfirmed Prediabetes
Source: PLoS One. 2016 Jul 18;11(7):e0157729. doi: 10.1371/journal.pone.0157729 (PMC4948775; doi:10.1371/journal.pone.0157729)
Supplement: S2 Table — (DOCX) [file pone.0157729.s007.docx]

S2 Table: Baseline Characteristics

| Variables | **At-risk for Diabetes** n= 19,288 mean± SD or % | **Unconfirmed Prediabetes** n=13,005 mean± SD or % | **Confirmed Prediabetes** n=1,545 mean± SD or % | *p*-value |
| --- | --- | --- | --- | --- |
| **Demographics** |  |  |  |  |
| **Age at Study Enrollment, yrs** | 48.67±15.28 | 54.09± 11.99 | 54.10± 13.02 | <.001 |
| **Age categories, %** |  |  |  | <.001 |
| 18-29 | 15.06 | 3.65 | 5.31 |  |
| 30-39 | 17.03 | 9.43 | 9.71 |  |
| 40-49 | 18.65 | 21.04 | 17.99 |  |
| 50-59 | 21.84 | 32.11 | 31.00 |  |
| 60-69 | 18.69 | 24.93 | 24.40 |  |
| ≥ 70 | 8.73 | 8.84 | 11.59 |  |
| **Gender, %** |  |  |  | <.001 |
| Male | 51.01 | 59.38 | 48.87 |  |
| Female | 48.99 | 40.62 | 51.13 |  |
| **Race/ethnicity** |  |  |  | <.001 |
| White | 81.17 | 87.92 | 89.58 |  |
| Asian | 2.86 | 1.60 | 1.68 |  |
| Black | 2.31 | 0.57 | 0.39 |  |
| Hispanic | 3.88 | 0.65 | 0.58 |  |
| Other  Unknown | 4.36  5.42 | 1.85  7.41 | 1.29  6.47 |  |
| **Clinical Characteristics** |  |  |  |  |
| **Chronic Conditions, %** |  |  |  |  |
| High Blood Pressure | 37.40 | 31.13 | 35.15 | .0004 |
| Depression | 17.36 | 18.6 | 27.38 | <.001 |
| Coronary Heart Disease | 9.22 | 9.4 | 11.78 | <.001 |
| Congestive Heart Failure | 3.64 | 2.99 | 5.24 | <.001 |
| Atrial Fibrillation | 3.02 | 2.01 | 3.50 | <.001 |
| **Medication class, %** |  |  |  |  |
| Anti-hypertensive | 28.31 | 26.91 | 27.83 | <.001 |
| Anti-neuroleptics | 2.59 | 1.94 | 6.60 | <.001 |
| Metformin | 1.17 | 1.96 | 3.56 | <.001 |
| Statin | 15.09 | 24.94 | 26.08 | <.001 |
| **Weight at baseline, kg** | 93.01±23.39 | 94.50±24.18 | 94.25±23.34 | <.001 |
| **BMI at baseline, %** |  |  |  | <.001 |
| <30 kg/m^2^ | 47.86 | 33.99 | 33.07 |  |
| ≥30 kg/m^2^ | 52.14 | 66.01 | 66.93 |  |
| **Follow-up time, yrs** | 5.23 | 5.14 | 4.94 | <.001 |
