# Supplementary material for: Incidental Risk of Type 2 Diabetes Mellitus among Patients with Confirmed and Unconfirmed Prediabetes
Source: PLoS One. 2016 Jul 18;11(7):e0157729. doi: 10.1371/journal.pone.0157729 (PMC4948775; doi:10.1371/journal.pone.0157729)
Supplement: S3 Table — (DOCX) [file pone.0157729.s008.docx]

**S3 Table: Incidental risk of type 2 diabetes mellitus** among patients with confirmed and unconfirmed prediabetes as compared to those at-risk for disease

|  | Discrete Survival Analysis Model | | | | |
| --- | --- | --- | --- | --- | --- |
| Covariates | # pts | # with Type 2 Diabetes Mellitus | Hazard Ratio | 95% Confidence Interval | *p*-value |
| **Demographics** |  |  |  |  |  |
| Study Group |  |  |  |  |  |
| At-risk for Diabetes | 19,288 | 1,190 | ---- | ---- | ---- |
| Unconfirmed Prediabetes | 13,005 | 1,391 | 1.67 | 1.53, 1.83 | <.001 |
| Confirmed Prediabetes | 1,545 | 302 | 2.73 | 2.37, 3.15 | <.001 |
| Age (years) |  |  |  |  |  |
| 18-29 | 3,462 | 47 | ---- | ---- | ---- |
| 30-39 | 4,662 | 203 | 2.34 | 1.55, 3.52 | <.001 |
| 40-49 | 6,612 | 413 | 3.07 | 2.07, 4.56 | <.001 |
| 50-59 | 8,867 | 882 | 3.96 | 2.68, 5.85 | <.001 |
| 60-69 | 7,223 | 921 | 3.92 | 2.65, 5.80 | <.001 |
| ≥ 70 | 3,012 | 417 | 3.67 | 2.46, 5.48 | <.001 |
| Female sex | 15,523 | 1,392 | 0.91 | 0.83, 0.98 | .018 |
| Race |  |  |  |  |  |
| Caucasian | 28,474 | 2,598 | ---- | ---- | ---- |
| Asian | 786 | 64 | 2.24 | 1.74, 2.88 | <.001 |
| Black | 525 | 32 | 1.23 | 0.85, 1.77 | .278 |
| Hispanic | 841 | 8 | 0.84 | 0.38, 1.86 | .662 |
| Other | 1,102 | 79 | 1.53 | 1.19, 1.98 | .001 |
| Unknown | 2,110 | 102 | 1.11 | 0.89, 1.37 | .356 |
| **Clinical Characteristics** |  |  |  |  |  |
| Chronic Conditions |  |  |  |  |  |
| Depression | 6,190 | 646 | 0.85 | 0.77, 0.94 | .001 |
| Coronary Heart Disease | 3,183 | 374 | 1.10 | 0.96, 1.25 | .164 |
| Congestive Heart Failure | 1,173 | 142 | 0.91 | 0.74, 1.12 | .364 |
| Atrial Fibrillation | 899 | 82 | 0.73 | 0.57, 0.94 | .014 |
| High Blood Pressure | 10,456 | 1,311 | 1.16 | 1.05, 1.27 | .002 |
| Medication Class |  |  |  |  |  |
| Anti-hypertension | 8,617 | 993 | 1.04 | 0.95, 1.15 | .370 |
| Atypical-neuroleptics | 854 | 85 | 0.86 | 0.68, 1.10 | .234 |
| Metformin | 535 | 190 | 4.01 | 3.37, 4.78 | <.001 |
| Statin | 6,556 | 746 | 0.96 | 0.87, 1.06 | .416 |
| BMI at baseline |  |  |  |  |  |
| <30 kg/m^2^ | 14,162 | 646 | ---- | ---- | ---- |
| ≥30kg/m^2^ | 19,676 | 2,237 | 1.98 | 1.76, 2.22 | <.001 |
|  | | | | | |
