# Supplementary material for: Incidental Risk of Type 2 Diabetes Mellitus among Patients with Confirmed and Unconfirmed Prediabetes
Source: PLoS One. 2016 Jul 18;11(7):e0157729. doi: 10.1371/journal.pone.0157729 (PMC4948775; doi:10.1371/journal.pone.0157729)
Supplement: S4 Table — (DOCX) [file pone.0157729.s009.docx]

**S4 Table: Incidental risk of type 2 diabetes mellitus associated with the study group and age greater or less 45 years of age among study patients.**

|  | Discrete Survival Analysis Model | | | | |
| --- | --- | --- | --- | --- | --- |
|  |  |  |  |  |  |
| Independent Variables | #pts | # with Type 2 Diabetes Mellitus | Hazard Ratio | 95% Confidence Interval | *p*-value |
| **Demographics** |  |  |  |  |  |
| Exposure Group |  |  |  |  |  |
| At-risk for Diabetes and <45 years | 7,828 | 238 | ---- | ---- | ---- |
| At-risk for Diabetes and ≥45 years | 11,460 | 1,013 | 1.86 | 1.54, 2.25 | <.010 |
| Unconfirmed Prediabetes and <45 years | 2,810 | 185 | 2.32 | 1.81, 2.99 | <.001 |
| Unconfirmed Prediabetes and ≥45 years | 10,195 | 1,308 | 2.90 | 2.35, 3.57 | <.001 |
| Confirmed Prediabetes and <45 years | 356 | 38 | 3.70 | 2.37, 5.77 | <.001 |
| Confirmed Prediabetes and ≥45 years | 1,189 | 290 | 5.23 | 4.16, 6.58 | <.001 |
| Female sex | 15,523 | 1,392 | 1.00 | 0.86, 1.16 | .978 |
| Race |  |  |  |  |  |
| White | 28,474 | 2,598 | ---- | ---- | ---- |
| Asian | 786 | 64 | 1.69 | 0.92, 3.11 | .093 |
| Black | 525 | 32 | 1.13 | 0.74, 1.73 | .560 |
| Hispanic | 841 | 8 | 0.78 | 0.25, 2.42 | .670 |
| Other | 1,102 | 79 | 1.46 | 1.07, 1.97 | .016 |
| Unknown | 2,110 | 102 | 1.24 | 0.98, 1.57 | .074 |
| **Clinical Characteristics** |  |  |  |  |  |
| Chronic Conditions |  |  |  |  |  |
| Depression | 6,190 | 646 | 0.87 | 0.76, 0.99 | .042 |
| Coronary Heart Disease | 3,183 | 374 | 1.22 | 1.06, 1.42 | .007 |
| Congestive Heart Failure | 1,173 | 142 | 0.95 | 0.76, 0.99 | .042 |
| Atrial Fibrillation | 899 | 82 | 0.71 | 0.54, 0.93 | .014 |
| High Blood Pressure | 10,456 | 1,311 | 1.14 | 1.01, 1.29 | .043 |
| Medication Class |  |  |  |  |  |
| Anti-hypertension | 8,617 | 993 | 0.88 | 0.75, 1.03 | .114 |
| Atypical-neuroleptics | 854 | 85 | 0.32 | 0.10, 1.05 | .060 |
| Metformin | 535 | 190 | 3.78 | 3.09, 4.64 | <.001 |
| Statin | 6,556 | 746 | 1.09 | 0.94, 1.25 | .247 |
| BMI at baseline |  |  |  |  |  |
| <30 kg/m^2^ | 14,162 | 646 | ---- | ---- | ---- |
| ≥30kg/m^2^ | 19,676 | 2,237 | 1.98 | 1.77, 2.22 | <.001 |
